# Development and validation of a climate change version of the man-made disaster-related distress scale (CC-MMDS)

**DOI:** 10.1016/j.joclim.2024.100356

**Published:** 2024-10-18

**Authors:** Jil Beckord, Julia Barbara Krakowczyk, Nadja Gebhardt, Leonie Sophie Geiser, Katharina Kamler, Christoph Nikendei, Eva-Maria Skoda, Martin Teufel, Alexander Bäuerle

**Affiliations:** a Clinic for Psychosomatic Medicine and Psychotherapy, University of Duisburg-Essen, LVR-University Hospital Essen, Essen, Germany; b Center for Translational Neuro- and Behavioral Sciences (C-TNBS), University of Duisburg-Essen, Essen, Germany; c Clinic for General Internal Medicine and Psychosomatic, University Hospital Heidelberg, Heidelberg, Germany

**Keywords:** Climate change, Man-made disasters, Psychological distress, Scale construction, Validation

## Abstract

**Introduction:**

Climate change, considered as a pressing man-made disaster, poses a fundamental threat to global health, with significant psychological consequences. However, measurement instruments assessing these consequences of climate change remain limited. This study aimed to address this gap by developing and validating an adapted version of the Man-Made Disaster-Related Distress Scale (MMDS), specifically designed for the assessment of indirect climate change-related psychological consequences.

**Materials and Methods:**

The items of the MMDS were adapted to climate change. The resulting “Climate Change – Man-Made Disaster-Related Distress Scale” (CC-MMDS), comprising 16 items, was validated in an online-based survey in Germany. Initially, 1070 participants were engaged in the survey.

**Results:**

The data of *n* = 715 participants was used for analysis. Exploratory and confirmatory factor analyses revealed a two-factor structure, encompassing “psychological distress” and “change of existing belief systems”, with acceptable to good model fit. The CC-MMDS demonstrated excellent reliability and good validity, with measurement invariance across gender, age, and educational level.

**Discussion:**

This study validated the CC-MMDS, confirming a two-factor structure. The scale demonstrated strong measurement properties, making it a promising standardized instrument for assessing climate change-related distress.

**Conclusion:**

With its strong psychometric properties and efficient administration, the CC-MMDS offers valuable insights for research and can aid mental health providers in identifying and supporting individuals impacted by climate change.

## Highlights

• Development of a man-made climate change-related distress scale.
• The resulting CC-MMDS^1^ consists of an economic 16-item scale.
• First evaluation revealed good psychometric properties.
• The CC-MMDS exerts scalar invariance across gender, age, and educational level.

## 1 Introduction

Disasters, both natural (e.g., hurricanes) and man-made (e.g., war), have a significant negative impact on human life and health worldwide [1,2]. As human activities are recognized as the main cause of global warming and climate change (CC) [3,4], climate change can consequently be categorized as a man-made disaster and is commonly referred to as “man-made climate change” [5]. With its significant consequences on mental health, CC poses a fundamental public health threat, necessitating action [6,7].

There are direct and indirect pathways through which CC can impact mental health negatively [[7], [8], [9]]. On the one hand, extreme weather events and rising temperatures directly influence mental well-being and are associated with an increase in psychological disorders [9], like depression, anxiety, and especially post-traumatic stress disorder [[10], [11], [12], [13]]. Moreover, increasing or high temperatures were found to be correlated with higher suicide rates and more aggressive behavior [[14], [15], [16]]. On the other hand, CC affects mental health indirectly through the awareness of present and future consequences of CC [9], leading to heightened psychological distress and negative emotional states like anxiety, worry, anger or guilt [[17], [18], [19]]. As a result, the direct and indirect pathways of CC have multifactorial negative psychological consequences, emphasizing the relevance of this issue in psychotherapeutic contexts [20].

Despite the acknowledged adverse impacts and urgent calls for action from organizations such as the World Health Organization [7] and United Nations [3], research on measurement instruments assessing the mental health consequences of man-made CC is still limited [4]. There are several instruments that assess the general perception or awareness of CC [[21], [22], [23]], some instruments that assess solastalgia [24,25], and some that measure the mental health consequences of CC in form of distress, worries, or anxiety [[26], [27], [28], [29], [30]]. While the Climate Change Anxiety Scale (CCAS) [26] represents one such instrument, it has faced criticism, particularly concerning validity and factor structure [31]. Moreover, the CCAS has been criticized for its high item difficulties, resulting in subjectively highly distressed individuals receiving low scores, thereby rendering the CCAS unsuitable to identify affected individuals [32]. To address these shortcomings, other instruments like the Eco-Anxiety Questionnaire (EAQ-22) [30] and the Climate Change Distress and Impairment Scale (CC-DIS) [27] were developed. However, those existing questionnaires assess only one aspect of CC-related psychological consequences, like anxiety or distress, while neglecting the man-made aspect and the associated doubts in belief systems. A change in belief systems has been previously demonstrated to display a significant factor, next to psychological distress, contributing to distress associated with man-made disasters [33]. It is particularly important to assess this construct in the context of CC, as anthropogenic actions are recognized as the main cause of CC, labeling it as a man-made disaster [3].

The need for suitable measurement instruments led to the development of the “Climate Change – Man-Made Disaster-Related Distress Scale” (CC-MMDS), adapted from the validated “Man-Made Disaster-Related Distress Scale” (MMDS) [33]. Existing measurement instruments targeting the mental health consequences of man-made disasters in general present a viable foundation for an adaptation to the specific context of man-made CC. The MMDS, validated in 2022, has shown promising measurement properties and results in assessing psychological distress from man-made disasters.

The CC-MMDS aims to capture the multifaceted impacts and emotional responses associated with CC, like distress, anxiety, anger, worries, and doubts in belief systems. Based on the MMDS [33], the CC-MMDS was hypothesized to have a two-factor structure, good validity and reliability, and measurement invariance across gender, age, and education.

## 2 Material and methods

This study adheres to the STROBE guidelines for cross-sectional studies [34], received ethical approval from the University of Duisburg-Essen (23–11,342-BO), and was preregistered at Open Science Framework (OSF.IO/B5NAK) [35].

### 2.1 Development of the climate change – man-made disaster-related distress scale

The CC-MMDS, adapted from the original 14-item MMDS [33], was developed through a literature review by three independent researchers to ensure content validity. Based on the literature review, the original MMDS items were adjusted for CC context and extended by three new items capturing feelings of guilt, anger, and doubts about the political approach to tackle CC, as these were feelings often associated with CC. An interdisciplinary panel of four psychologists and two physicians, all experienced in public and mental health research [9,36,37], evaluated and refined the items through multiple feedback rounds. In each round, feedback was collected, revisions were made, and the updated version was reviewed again until consensus was reached.

The initial CC-MMDS consisted of 17 items (Appendix A), each to be rated on a 7-point Likert scale ranging from 1 = "strongly disagree" to 7 = "strongly agree" (other values not specified). Higher scores indicate higher CC-related distress. One item was excluded after item analysis due to its poor discrimination and low difficulty (Appendix B). The final CC-MMDS comprises 16 items (Appendix C).

### 2.2 Participants and procedure

The sample comprised 1070 German-speaking individuals, aged ≥ 18, who voluntarily participated in the anonymous cross-sectional study from October 2023 to February 2024. The survey was administered using the online software Unipark [38] and disseminated across public channels (personal and professional contacts, social media, flyers). Electronic informed consent was obtained from all participants.

805 (75.23 %) participants successfully completed the survey. 15 participants aged under 18 were excluded, along with 32 participants who failed instructed response items and 43 with longstring responding (> 15) [39]. The remaining 715 participants were included in all subsequent analyses. The sample size met recommended guidelines for validation studies [40].

### 2.3 Measures

Sociodemographic information, such as gender, age, educational level, and net income were assessed. Additionally, participants were asked to indicate how they would position themselves with regard to CC (I see myself “strongly as a CC denier” / “more as a CC denier” / “neutral/neither” / “more as a CC activist” / “strongly as a CC activist”).

The following standardized assessment instruments were applied to test for validity of the CC-MMDS. Further details regarding the validation process are provided under Section 2.4.

*Short scale for Environmental Awareness* (EA) [41]. This scale measures environmental awareness (α = .80) on two subscales: environmental affect and cognition (6 items, α = .78) and environmental behavior (3 items, α = .54). The items can be answered on a 4-point and 6-point Likert scale, respectively.

*Climate Change Anxiety Scale* (CCAS) [26]. The CCAS measures the emotional response to CC (α = .91) on two subscales: cognitive-emotional impairment (8 items, α = .84), and functional impairment (5 items, α = .88). The German version consists of 13 items that can be answered on a 7-point Likert scale [42].

*Sense of Coherence Scale Short Form* (SOC-L9) [43]. The unidimensional SOC-L9 measures sense of coherence (α = .86) and is a revised short form of the sense of coherence scale (SOC) by Antonovsky [44]. It consists of nine items that are based on a 7-point Likert scale.

*Big Five Inventory-10* (BFI-10) [45]. The BFI-10 is a questionnaire that assesses personality traits (α = .56) based on the OCEAN model: openness (*r*_SB_ = .54), conscientiousness (*r*_SB_ = .45), extraversion (*r*_SB_ = .77), agreeableness (*r*_SB_ = .38), and neuroticism (*r*_SB_ = .63). Each trait is represented by two items answered on a 5-point Likert scale.

*Generalized Anxiety 7-item Scale* (GAD-7) [46]. The GAD-7 assesses anxiety symptoms (α = .89). It consists of seven items that can be answered on a 4-point Likert scale.

*Patient Health Questionnaire-8* (PHQ-8) [47]. The PHQ-8 is a screening instrument to assess depression symptoms (α = .88). It consists of eight items and is based on a 4-point Likert scale.

*Distress Thermometer* (DT) [48]. The DT measures psychological distress on one visual scale from 0 to 10.

### 2.4 Data analysis

Data analysis was carried out in four sequential steps using R version 4.2.2 [49]:

In the first step, an exploratory factor analysis (EFA) was performed. Item suitability was evaluated with the Kaiser-Meyer-Olkin (KMO) measure and Bartlett's test of sphericity. The underlying factor structure was determined by plotting the Eigenvalues via a scree test, inspecting the very simple structure criterion (VSS), and Velicer's minimum average partial (MAP) criterion [50,51]. Subsequent factor extraction was conducted using maximum likelihood estimation with promax rotation.

In the second step, a confirmatory factor analysis (CFA) was conducted to confirm the model from the preceding EFA using the R package lavaan [52] with maximum likelihood robust (MLR) estimator. Model fit was evaluated as good with a Comparative Fit Index (CFI) and Tucker Lewis Index (TLI) > .90, a Root Mean Square Error of Approximation (RMSEA) < .06, and a Standardized Root Mean Square Residual (SRMR) < .08 [53].

In the third step, reliability and discriminant, convergent, and criterion validity of the CC-MMDS and its subscales were tested. Reliability was evaluated with Cronbach's α and McDonald's ω. In order to verify convergent validity, Pearson correlations between CC-MMDS, its subscales, and other related scales (subscales of EA and CCAS, SOC-L9, BFI-10 neuroticism) were examined. Discriminant validity was assessed by correlating the CC-MMDS and its subscales with the remaining BFI-10 subscales. Criterion validity was examined through correlations with GAD-7, PHQ-8, and DT.

In the fourth step, the final model was tested regarding measurement invariance across gender, educational level, and age. For gender, a female (*n* = 473, 66.15 %) and a male (*n* = 227, 31.75 %) group were compared. 15 persons that identified as inter/diverse were excluded from this analysis. Regarding educational invariance, the study sample was divided into the three groups: university degree (*n* = 424, 59.30 %), university entrance qualification (highest school certificate in Germany after 13 years of education; *n* = 221, 30.91 %), and other school certificate (*n* = 70, 9.80 %). To assess age invariance, participants were categorized into three age groups: 19–30 years (*n* = 309, 43.22 %), 31–50 years (*n* = 184, 25.73 %), and 51–87 years (*n* = 212, 29.65 %). Measurement invariance was tested with three consecutive steps – configural, metric, and scalar invariance – of multiple-group CFA [54,55]. Invariance was defined as ΔCFI < 0.01 and ΔRMSEA < 0.015 [54,55].

## 3 Results

### 3.1 Sample characteristics

The study sample (*N* = 715) had a mean age of 39.14 (± 15.59) years, with an age range between 18 and 87 years. Additional characteristics of the study sample are detailed in Table 1.

**Table 1:** Demographic characteristics of the study sample (*N* = 715).

Categorical variables	*n* (%)
Gender	
Female	473 (66.15)
Male	227 (31.75)
Inter/divers	15 (2.1)
Educational level	
University degree	424 (59.3)
University entrance qualification^a^	221 (30.9)
Upper secondary school^b^	43 (6.0)
Lower secondary school^c^	14 (2.0)
Other	13 (1.8)
Net income (per month)	
No answer	48 (6.71)
< 1000,- Eur	170 (23.78)
1000–3000,- Eur	307 (42.94)
3000–5000,- Eur	146 (20.42)
> 5000,- Eur	44 (6.15)
Personal positioning with regard to climate change	
Strongly as a climate change denier	3 (0.42)
More as a climate change denier	6 (0.84)
Neutral/neither	212 (29.65)
More as a climate change activist	376 (52.59)
Strongly as a climate change activist	118 (16.50)

a 13 years of education.

b 10 years of education.

c mandatory 9 years of education.

### 3.2 Step one – exploratory factor analysis

The KMO test indicated excellent sampling adequacy with a value of 0.95 [56]. Bartlett's Test of Sphericity was significant, *Χ*²(120) = 7822.81, *p* < .001 [57]. Analysis of the scree plot and VSS criterion suggested a two-factor structure. VSS complexity 2 reached a maximum of 0.96, while Velicer's MAP criterion reached a minimum of 0.02 with two factors. These factors explained 58 % of cumulative variance. Based on these results, a factor structure with two inter-correlating underlying factors was proposed.

**Fig. 1:**
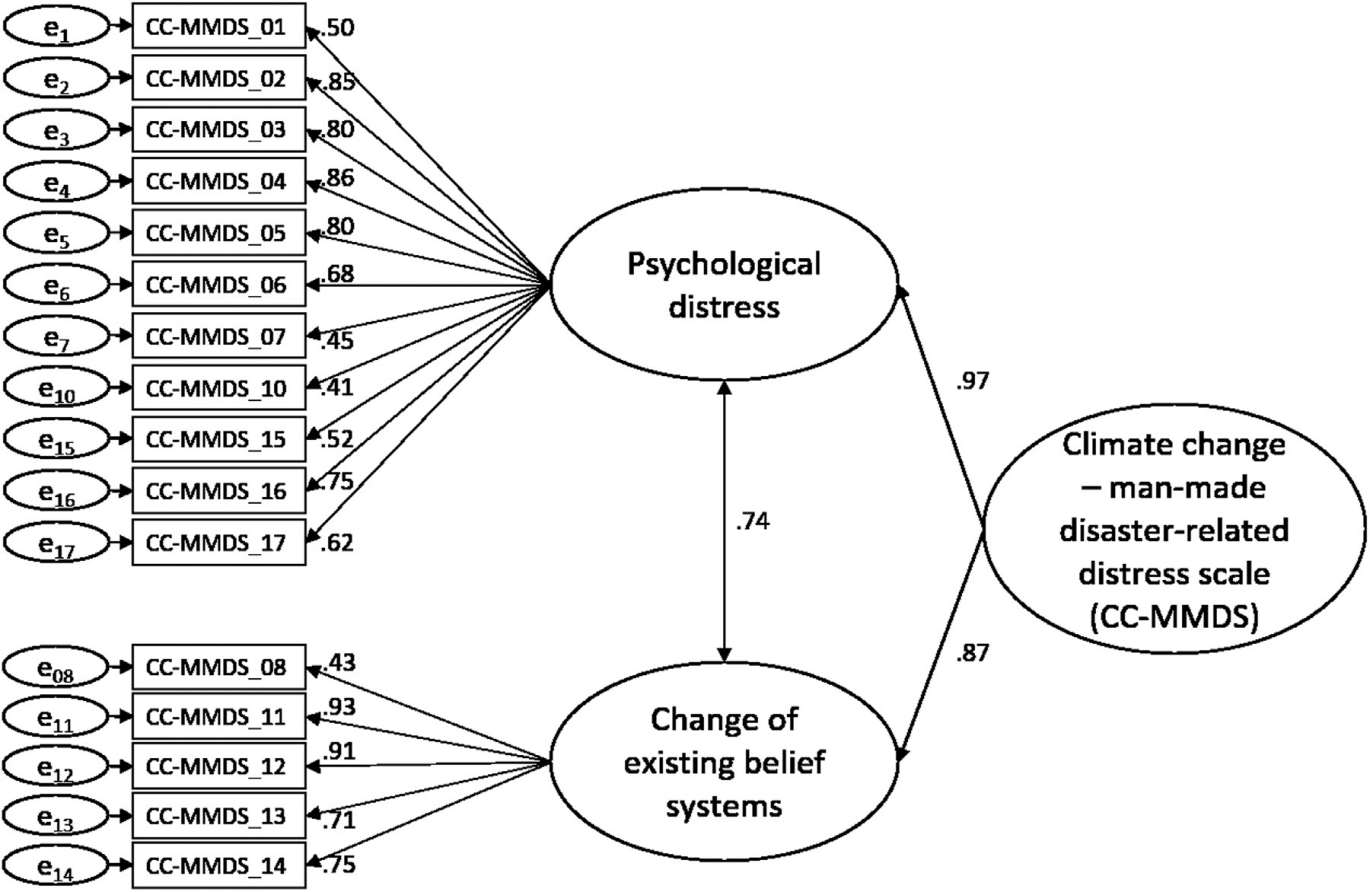
Factor structure of the CC-MMDS with associated factor loadings.

The first factor includes items related to negative emotional states, including distress, anxiety, and depression. The second factor involves items related to doubts about the future, social norms and values, and the political approach to CC. Based on item content and the MMDS [26], the two factors were termed “psychological distress” and “change of existing belief systems”. Fig. 1 and Appendix D provide detailed factor loadings.

### 3.3 Step two – confirmatory factor analyses

Based on the EFA results, a two-factor model of the CC-MMDS was tested with CFA (Model 1). Items 1–7, 10, and 15–17 were assigned to the first factor, and items 8 and 11–14 to the second factor. Another pre-considered theoretical model was tested where items 8 and 10 were switched based on their content (Model 2). A third model was tested where items 7, 8, and 10 were excluded due to low factor loadings (Model 3).

Results of the CFA are presented in Table 2. Despite Model 3 showing the best model fit indices, Model 1 was chosen for subsequent analyses as there were only marginal differences and in order to retain as much information as possible [58].

**Table 2:** Results of CFA.

Model	Chi-square	df	CFI	TLI	RMSEA	SRMR	AIC	SABIC
Model 1	681.431	103	.913	.898	.096	.053	39,565.85	39,611.96
Model 2	715.681	103	.907	.892	.099	.059	39,608.17	39,654.28
Model 3	485.863	64	.925	.908	.104	.049	31,743.84	31,781.56

Note: df, degrees of freedom; CFI, Comparative Fit Index; TLI, Tucker Lewis Index; RMSEA, Root Mean Square Error of Approximation; SRMR, Standardized Root Mean Square Residual; AIC, Akaike Information Criterion; SABIC, Sample-size Adjusted Bayesian Information Criterion.

### 3.4 Climate change – man-made disaster-related distress scale (CC-MMDS)

The mean score of the CC-MMDS was 4.28 (± 1.37, range 1–7). The CC-MMDS was divided into two subscales: “psychological distress” (feelings of depression, anxiety, and distress related to CC) and “change of existing belief systems” (doubts about the future, social norms and values, and the political system's approach to CC). Participants averaged 3.96 (± 1.45) on psychological distress, and 4.98 (± 1.48) on change of existing belief systems.

### 3.5 Step three – reliability and validity analyses

Reliability analysis indicated a high internal consistency of the 16-item two-factor CC-MMDS, with Cronbach's α = .94 and McDonald's ω = .95. The psychological distress subscale had α = .93 and ω = .93, and the change of existing belief systems subscale had α = .88 and ω = .88.

Convergent validity analyses revealed that the CC-MMDS and its subscales had highly significant positive correlations with the validated CC subscales of the EA and CCAS, highly significant negative correlation with the SOC-L9, and a significant positive correlation with the BFI-10 neuroticism scale.

Discriminant validity analyses showed no correlations of the CC-MMDS and its subscales with the extraversion and agreeableness subscales of the BFI-10. There was a significant negative correlation between the conscientiousness subscale and both the CC-MMDS and change of existing belief systems subscale. There was also a significant positive correlation between the openness subscale and both the CC-MMDS and psychological distress subscale.

Criterion validity analyses showed highly significant positive correlations between the CC-MMDS and its subscales and the GAD-7, PHQ-8, and DT. Results of the validation analyses are depicted in Table 3.

**Table 3:** Correlation coefficients and significance levels of the CC-MMDS and its subscales.

Scales	CC-MMDS	Psychological distress	Change of belief systems
CC-MMDS	1.00	0.97***	0.87***
CC-MMDS-PD	0.97***	1.00	0.74***
CC-MMDS-CBS	0.87***	0.74***	1.00
Convergent Validity			
EA-AC	0.61***	0.57***	0.60***
EA-B	0.34***	0.32***	0.34***
CCAS-CEI	0.63***	0.66***	0.47***
CCAS-FI	0.59***	0.60***	0.48***
SOC-L9	−0.25***	−0.27***	−0.19***
BFI-N	0.17**	0.19**	0.10**
Discriminant Validity			
BFI-E	−0.04	−0.05	−0.04
BFI-C	−0.08*	−0.07	−0.10**
BFI-O	0.08*	0.08*	0.05
BFI-A	−0.04	−0.04	−0.05
Criterion Validity			
GAD-7	0.36***	0.39***	0.26***
PHQ-8	0.31***	0.32***	0.23***
DT	0.28***	0.29***	0.21***

Note: *p < .05, **p < .01, ***p < .001. CC-MMDS-, climate change – man-made disaster-related distress scale-(psychological distress, change of existing belief systems); EA-, Environmental Awareness-(Affect and Cognition, Behavior); CCAS-, Climate Change Anxiety Scale-(Cognitive Emotional Impairment, Functional Impairment); SOC-L9, Sense of Coherence Scale; BFI-, Big Five Inventory-(Neuroticism, Extraversion, Conscientiousness, Openness, Agreeableness); GAD-7, General Anxiety Disorder-7; PHQ-8, Patient Health Questionnaire-8; DT, Distress Thermometer.

### 3.6 Step four – measurement invariance

Based on the accepted two-factor model from CFA, measurement invariance of the CC-MMDS was tested across gender, age, and educational level. Results in Table 4 indicate minimal deterioration in model fit from weak to strong invariance, with ΔCFI < .01 and ΔRMSEA < .015, establishing scalar invariance across these factors.

**Table 4:** Measurement invariance testing.

Model	Chi-square	df	CFI	TLI	RMSEA	SRMR	AIC	SABIC	ΔCFI	ΔRMSEA
	681.431	103	.913	.898	.096	.053	39,565.85	39,611.96		
Gender										
Configural	793.762	206	.911	.896	.096	.054	38,776.12	38,910.96	−0.002	.000
Metric	817.856	220	.910	.902	.094	.061	38,766.99	38,882.57	−0.001	−0.002
Scalar	855.579	234	.907	.905	.092	.063	38,775.43	38,871.74	−0.003	−0.002
Age										
Configural	918.632	309	.910	.896	.097	.056	39,060.64	39,263.93	−0.003	.001
Metric	971.155	337	.908	.902	.095	.069	39,050.88	39,215.45	−0.002	−0.002
Scalar	1038.193	365	.903	.904	.093	.071	39,061.18	39,187.04	−0.005	−0.002
Education										
Configural	984.683	309	.904	.888	.101	.056	39,687.35	39,892.72	−0.009	.005
Metric	1018.324	337	.905	.898	.096	.062	39,653.06	40,197.17	.001	−0.005
Scalar	1065.163	365	.903	.904	.093	.063	39,638.54	39,765.67	−0.002	−0.003

Note: df, degrees of freedom; CFI, Comparative Fit Index; TLI, Tucker Lewis Index; RMSEA, Root Mean Square Error of Approximation; SRMR, Standardized Root Mean Square Residual; AIC, Akaike Information Criterion; SABIC, Sample-size Adjusted Bayesian Information Criterion; ΔCFI, change in Comparative Fit Index; ΔRMSEA, change in RMSEA.

## 4 Discussion

This study aimed to develop and validate an instrument specifically designed to capture psychological consequences of CC. Despite increasing concerns in psychotherapeutic settings about CC-related distress among patients, psychotherapists often feel inadequately prepared to address these issues [59]. Given the limited research and measurement instruments in this area, the CC-MMDS can gain new insights and enhance care.

The EFA confirmed a two-factor structure for the CC-MMDS, consistent with the original MMDS. Accordingly, these factors were labeled as 1) psychological distress and 2) change of existing belief systems. The psychological distress factor encompasses emotions commonly associated with CC, such as anxiety, depression, distress, worries, helplessness, guilt, and uncertainty [10,11,17,19]. Meanwhile, the change of existing belief systems factor assesses doubts about personal values, social beliefs, norms, and structures, a just world, and the political approach to CC. This is as CC is often associated with the reevaluation of personal values and a subsequent change towards climate-friendly behavior, and with a loss of trust in the political system to handle CC [60,61]. The unexpected switch of items 08 and 10 by the EFA can be theoretically justified: Item 10 might have been re-assigned because the re-evaluation of the one's worldview is often accompanied by uncertainty and anxiety [61]. Item 08 might have been re-assigned because anger has a different emotional quality than other emotions, motivating action [62] and correlating with doubts regarding political agents, perceptions of justice, or mankind [60].

Subsequent CFA further confirmed the two-factor structure, testing three models with different item allocations. Model 1, based on EFA outcomes, showed superior fit compared to Model 2, which involved reassigning two items based on their content. Model 1 was preferred over Model 3, which excluded three items due to low factor loadings and demonstrated a slightly better fit, as marginal differences in model fit indices and the potential loss of relevant content favored retaining all items [58]. Specifically, items 08 (anger) and 07 (guilt), are both commonly reported in association with CC [19,63]. Model 1 of the CFA demonstrated an acceptable-to-good fit: Both the CFI and TLI indicated a good fit [53] and the SRMR also met the recommended threshold. Although RMSEA did not meet guidelines [53], it remained marginal [64].

Reliability analyses revealed excellent internal consistencies for the CC-MMDS and its two subscales, while validity analyses showed overall good results. Significant positive correlations between the CC-MMDS scales and other CC scales confirmed convergent validity. Moreover, there was a small yet significant positive correlation of the CC-MMDS and its subscales with the BFI-10 neuroticism subscale and a significant negative correlation with the SOC-L9. This is in line with previous validity analyses of the MMDS and further supports convergent validity.

Discriminant validity was supported by the absence of correlations between the CC-MMDS scales and the extraversion and agreeableness subscales of the BFI-10. However, there was a significant negative correlation between the CC-MMDS and its change of existing belief systems subscale with the conscientiousness subscale of the BFI-10, likely due to conscientiousness mitigating beliefs in a just world [65]. Additionally, a significant positive correlation was found between the CC-MMDS and its psychological distress subscale with the BFI-10 openness subscale, suggesting that greater openness leads to less denial and more concerns about CC [66].

Finally, criterion validity was suggested by highly significant correlations between the CC-MMDS and the GAD-7, PHQ-8, and DT, indicating that CC-MMDS captures symptoms of anxiety, depression, and distress, aligning with previous MMDS validations. The low correlation indices emphasize good criterion validity, as they indicate that the CC-MMDS measures CC-specific distress distinct from general psychological distress [62].

Measurement invariance across gender, age, and educational level was examined to ensure accurate interpretation of group differences, a critical aspect of measurement instruments [67]. Scalar invariance was confirmed for the CC-MMDS, indicating that it measures equally across women and men, age groups, and different educational levels. This contrasts with the MMDS, which showed limited invariance, potentially influenced by differences in sample sizes.

Some limitations should be noted. Firstly, a potential selection bias may exist due to the convenience sample, overrepresenting women and individuals with a university degree [68,69]. Additionally, CC was treated as a man-made disaster, despite some people being skeptical about humanity's responsibility in CC [70]. Moreover, the sample was unbalanced regarding CC position, with a majority identifying as CC activists and only a few as deniers, possibly resulting from a social desirability bias [70]. Future studies should address the social desirability bias, e.g. through a more nuanced language like “skepticism” instead of “denial” and use a population-representative sample to enhance generalizability. As the CC-MMDS was validated only in a German-speaking region, international validation is needed to explore cross-regional differences and comparisons between directly and indirectly affected regions of CC. Furthermore, the cross-sectional design limits the ability to capture long-term developments and consequences of CC. Consequently, future research should evaluate the CC-MMDS's predictive validity within a longitudinal study.

Despite its’ limitations and given the limited research on CC and mental health, the CC-MMDS offers new insights on the multifaceted nature of CC's negative mental health impacts. Moving forward, the CC-MMDS can help mental health professionals identify individuals in need of psychosocial support due to CC-related distress. This topic has become increasingly relevant, which is further highlighted by the recent world health assembly of the WHO in 2024, which has primarily dealt with the strengthening of mental health and psychosocial support regarding natural and man-made disasters [71]. To the best of our knowledge, the CC-MMDS is the first measurement instrument that acknowledges CC´s man-made nature and thereby captures CC-related distress not only as a type of psychological distress but also as a change in existing belief systems, leading to a more holistic picture. This is particularly promising for clinical practice, as it helps mental health professionals to assess which of the two factors (psychological distress or change in belief systems) displays a higher burden for their patients and to provide tailored psychotherapeutic support. This is important, as research has shown that psychotherapists often feel inadequately prepared to address CC-related issues [59]. Ultimately, it can guide the development of targeted interventions for adapting to CC challenges.

## 5 Conclusion

Man-made CC, like other man-made disasters, has significant mental health consequences. However, reliable measurement tools are limited. Addressing this gap, the CC-MMDS was developed to specifically assess CC-related distress. Comprising 16 items distributed across two subscales – psychological distress and change of existing belief systems – the CC-MMDS demonstrates excellent reliability and good validity. Additionally, it exhibits measurement invariance across gender, age, and educational level. Thus, the CC-MMDS can serve as a standardized measurement instrument within CC research. Scientifically, it offers unprecedented insights into CC-related distress and its potential connections to mental health disorders. In the future, the CC-MMDS will aid mental health providers in quickly identifying individuals in need of psychosocial support due to CC. Moreover, it promises to spearhead the development of targeted interventions tailored to foster resilience amidst the challenges posed by CC.

## Data statement

The data is available from the CA on reasonable request.

## Ethical standards

This study was approved by the ethical committee of the medical faculty of the University of Duisburg-Essen (23–11,342-BO).

## CRediT authorship contribution statement

**Jil Beckord:** Writing – review & editing, Writing – original draft, Visualization, Validation, Project administration, Methodology, Investigation, Funding acquisition, Formal analysis, Data curation, Conceptualization. **Julia Barbara Krakowczyk:** Writing – review & editing, Writing – original draft, Project administration, Methodology, Investigation, Conceptualization. **Nadja Gebhardt:** Writing – review & editing, Methodology, Conceptualization. **Leonie Sophie Geiser:** Writing – review & editing, Writing – original draft, Investigation, Formal analysis, Data curation. **Katharina Kamler:** Writing – review & editing, Investigation. **Christoph Nikendei:** Writing – review & editing, Methodology, Conceptualization. **Eva-Maria Skoda:** Writing – review & editing, Validation, Supervision, Project administration, Methodology, Investigation, Funding acquisition, Conceptualization. **Martin Teufel:** Writing – review & editing, Validation, Supervision, Project administration, Methodology, Investigation, Conceptualization. **Alexander Bäuerle:** Writing – review & editing, Validation, Supervision, Project administration, Methodology, Investigation, Funding acquisition, Conceptualization.

## Declaration of competing interest

The authors declare the following financial interests/personal relationships which may be considered as potential competing interests:

Alexander Baeuerle reports financial support was provided by Drs. Graute and Graute-Oppermann-Foundation. If there are other authors, they declare that they have no known competing financial interests or personal relationships that could have appeared to influence the work reported in this paper.

## Appendix Supplementary materials

Image, application 1

Image, application 2

Image, application 3

## References

[1] Marshall J., Wiltshire J., Delva J., Bello T., Masys A.J. (2020). Natural and manmade disasters: vulnerable populations. Global health security: recognizing vulnerabilities, creating opportunities.

[2] Sederer L.I. (2012). Are human made disasters different?. Epidemiol Psychiatr Sci.

[3] United Nations. What is climate change, https://www.un.org/en/climatechange/what-is-climate-change [accessed 09.04.2024]

[4] Cuijpers P., Kumar M., Karyotaki E. (2023). Climate change and mental health—time to act now. JAMA Psych.

[5] Berger N., Lindemann A.K., Böl G.F. (2019). Wahrnehmung des Klimawandels durch die Bevölkerung und Konsequenzen für die Risikokommunikation. Bundesgesundheitsbl.

[6] IANPHI. IANPHI roadmap for action on health and climate change – engaging and supporting national public health institutes as key climate actors, https://ianphi.org/news/2021/roadmap-climate-change.html;2021 [accessed 09.04.2024]

[7] (2023). https://www.who.int/news-room/fact-sheets/detail/climate-change-and-health.

[8] Cianconi P., Betrò S., Janiri L. (2020). The impact of climate change on mental health: a systematic descriptive review. Front Psych.

[9] Gebhardt N., van Bronswijk K., Bunz M., Müller T., Niessen P., Nikendei C. (2023). Scoping review of climate change and mental health in Germany - direct and indirect impacts, vulnerable groups, resilience factors. J Health Monit.

[10] Clayton S. (2021). Climate change and mental health. Curr Environ Health Rep.

[11] Cunsolo A., Harper S.L., Minor K., Hayes K., Williams K.G., Howard C. (2020). Ecological grief and anxiety: the start of a healthy response to climate change?. Lancet Planet Heal.

[12] Otto K., Boos A., Dalbert C., Schöps D., Hoyer J. (2006). Posttraumatic symptoms, depression, and anxiety of flood victims: the impact of the belief in a just world. Pers Individ Dif.

[13] Reyes M.E.S., Carmen B.P.B., Luminarias MEP Mangulabnan S.A.N.B., Ogunbode C.A. (2023). An investigation into the relationship between climate change anxiety and mental health among Gen Z Filipinos. Curr Psychol.

[14] Eisele F., Flammer E., Steinert T., Knoblauch H. (2021). Aggressive incidents in psychiatric hospitals on heat days. BJPsych Open.

[15] Müller H., Biermann T., Renk S., Reulbach U., Ströbel A., Kornhuber J., Sperling W. (2011). Higher environmental temperature and global radiation are correlated with increasing suicidality – a localized data analysis. Chronobiol Int.

[16] Schneider A., Hampel R., Ladwig K.H., Baumert J., Lukaschek K., Peters A., Breitner S. (2020). Impact of meteorological parameters on suicide mortality rates: a case-crossover analysis in Southern Germany (1990–2006). Sci Total Environ.

[17] Grothmann T., Frick V., Harnisch R., Münsch M., Kettner S.E., Thorun C., Umweltbewusstsein in Deutschland (2022).

[18] Pihkala P. (2022). Toward a taxonomy of climate emotions. Front Clim.

[19] Stanley S.K., Hogg T.L., Leviston Z., Walker I. (2021). From anger to action: differential impacts of eco-anxiety, eco-depression, and eco-anger on climate action and wellbeing. J Clim Change Health.

[20] Gebhardt N., Saur C., Herrmann B., Friederich H.C., Nikendei C. (2023). Nun sag’, wie hast du's mit der Klimakrise?. Die Psychotherapie.

[21] Gönen Ç., Deveci E.Ü., Aydede M.N. (2023). Development and validation of climate change awareness scale for high school students. Environ Dev Sustain.

[22] Van Valkengoed A.M., Steg L., Perlaviciute G. (2021). Development and validation of a climate change perceptions scale. J Environ Psychol.

[23] Walker S., Mcneal K. (2013). Development and validation of an instrument for assessing climate change knowledge and perceptions: the climate stewardship survey (CSS). Int Electron J Environ Educ.

[24] Cáceres C., Leiva-Bianchi M., Serrano C., Ormazábal Y., Mena C., Cantillana J.C. (2022). What is solastalgia and how is it measured? sos, a validated scale in population exposed to drought and forest fires. Int J Env Res Pub He.

[25] Christensen B.K., Monaghan C., Stanley S.K., Walker I., Leviston Z., Macleod E., Rodney R.M., Greenwood L.M., Heffernan T., Evans O., Sutherland S., Reynolds J., Calear A.L., Kurz T., Lane J. (2024). The brief solastalgia scale: a psychometric evaluation and revision. EcoHealth.

[26] Clayton S., Karazsia B.T. (2020). Development and validation of a measure of climate change anxiety. J Environ Psychol.

[27] Hepp J., Klein S.A., Horsten L.K., Urbild J., Lane S.P. (2023). Introduction and behavioral validation of the climate change distress and impairment scale. Sci Rep.

[28] Stewart A.E. (2021). Psychometric properties of the climate change worry scale. Int J Env Res Pub He.

[29] Weiß M., Gutzeit J., Hein G. (2024). Development and validation of the domain-specific climate change distress scale. J Environ Psychol.

[30] Zeier P., Wessa M. (2024). Measuring eco-emotions: a German version of questionnaires on eco-guilt, ecological grief, and eco-anxiety. Discov Sustain.

[31] Mouguiama-Daouda C., Blanchard M.A., Coussement C., Heeren A. (2022). On the measurement of climate change anxiety: french validation of the climate anxiety scale. Psychol Belg.

[32] Schwartz S.E., Benoit L., Clayton S., Parnes M.F., Swenson L., Lowe S.R. (2023). Climate change anxiety and mental health: environmental activism as buffer. Curr Psychol.

[33] Krakowczyk J.B., Beckord J., Planert J., Kohl P., Schweda A., Teufel M., Bäuerle A. (2023). Development and psychometric evaluation of the man-made disaster-related distress scale (MMDS). Psych Res.

[34] Von Elm E., Altman D.G., Egger M., Pocock S.J., Gøtzsche P.C., Vandenbroucke J.P., STROBE Initiative (2007). Strengthening the reporting of observational studies in epidemiology (STROBE) statement: guidelines for reporting observational studies. BMJ.

[35] Beckord J., Krakowczyk J.B., Gebhardt N., Nikendei C., Teufel M., Bäuerle A. (2023). Development and validation of a climate change version of the man-made disaster-related distress scale (MMDScc). OSF.

[36] Bäuerle A., Schräpler L., Marsall M., Engelmann G., Knoll-Pientka N., Schüren L.C., Niedergethmann M., Robitzsch A., Skoda E.M., Hasenberg T., Teufel M. (2022). Development and validation of dietary behavior inventory-surgery (DBI-S) in the scope of international post-bariatric surgery guidelines and recommendations. Nutrients.

[37] Bäuerle A., Steinbach J., Schweda A., Beckord J., Hetkamp M., Weismüller B., Kohler H., Musche V., Dörrie N., Teufel M., Skoda E.M. (2020). Mental health burden of the COVID-19 outbreak in germany: predictors of mental health impairment. J Prim Care Commun Health.

[38] Tivian X.I. (2023). GmbH. Unipark, Hürth.

[39] Ward M.K., Meade A.W. (2023). Dealing with careless responding in survey data: prevention, identification, and recommended best practices. Annu Rev Psychol.

[40] Rouquette A., Falissard B. (2011). Sample size requirements for the internal validation of psychiatric scales. Int J Methods Psychiatr Res.

[41] Geiger S.M., Holzhauer B. (2020).

[42] Wullenkord M.C., Tröger J., Hamann K.R., Loy L., Reese G. (2021). Anxiety and climate change: a validation of the climate anxiety scale in a German-speaking quota sample and an investigation of psychological correlates. Clim Change.

[43] Schumacher J., Wilz G., Gunzelmann T., Brähler E. (2000). Die Sense of Coherence Scale von Antonovsky – Teststatistische Überprüfung in einer repräsentativen Bevölkerungsstichprobe und Konstruktion einer Kurzskala [Antonovsky's Sense of Coherence Scale–'Its validation in a population-based sample and the development of a new short scale]. PPmP: Psychother Psychosom Med Psychol.

[44] Antonovsky A. (1987).

[45] Rammstedt B., John O.P. (2007). Measuring personality in one minute or less: a 10-item short version of the Big Five Inventory in English and German. J Res Pers.

[46] Spitzer R.L., Kroenke K., Williams J.B., Löwe B. (2006). A brief measure for assessing generalized anxiety disorder: the GAD-7. Arch Intern Med.

[47] Kroenke K., Strine T.W., Spitzer R.L., Williams J.B., Berry J.T., Mokdad AH. (2009). The PHQ-8 as a measure of current depression in the general population. J Affect Disord.

[48] Mehnert A., Müller D., Lehmann C., Koch U. (2006). Die deutsche version des NCCN distress-thermometers: empirische Prüfung eines screening-instruments zur erfassung psychosozialer belastung bei krebspatienten. Z Psychiatr Psychol Psychother.

[49] Core Team R. (2022). https://www.R-project.org/.

[50] Revelle W., Rocklin T. (1979). Very simple structure: an alternative procedure for estimating the optimal number of interpretable factors. Multivar Behav Res.

[51] Velicer W.F. (1976). Determining the number of components from the matrix of partial correlations. Psychometrika.

[52] Rosseel Y. (2012). Lavaan: an R package for structural equation modeling. J Stat Softw.

[53] Hu L.T., Bentler P.M. (1999). Cutoff criteria for fit indexes in covariance structure analysis: conventional criteria versus new alternatives. Struct Equ Modeling.

[54] Hirschfeld G., Von Brachel R. (2014). Improving multiple-group confirmatory factor analysis in R–a tutorial in measurement invariance with continuous and ordinal indicators. Pract Assess Res Eval.

[55] Putnick D.L., Bornstein M.H. (2016). Measurement invariance conventions and reporting: the state of the art and future directions for psychological research. Dev Rev.

[56] Kaiser H.F., Rice J. (1974). Little jiffy, mark IV. Educ Psychol Meas.

[57] Bartlett M.S. (1951). The effect of standardization on a χ 2 approximation in factor analysis. Biometrika.

[58] Boateng G.O., Neilands T.B., Frongillo E.A., Melgar-Quiñonez H.R., Young S.L. (2018). Best practices for developing and validating scales for health, social, and behavioral research: a primer. Front Public Health.

[59] Trost K., Ertl V., König J., Rosner R., Comtesse H. (2024). Climate change-related concerns in psychotherapy: therapists’ experiences and views on addressing this topic in therapy. BMC Psychol.

[60] Gregersen T., Andersen G., Tvinnereim E. (2023). The strength and content of climate anger. Glob Environ Change.

[61] Lucas C., Leith P., Davison A. (2015). How climate change research undermines trust in everyday life: a review. Wiley Interdiscip Rev Clim Change.

[62] Contreras A., Blanchard M.A., Mouguiama-Daouda C., Heeren A. (2024). When eco-anger (but not eco-anxiety nor eco-sadness) makes you change! A temporal network approach to the emotional experience of climate change. J Anxiety Disord.

[63] Ágoston C., Csaba B., Nagy B., Kőváry Z., Dúll A., Rácz J., Demetrovics Z. (2022). Identifying types of eco-anxiety, eco-guilt, eco-grief, and eco-coping in a climate-sensitive population: a qualitative study. Int J Environ Res Public Health.

[64] Browne M.W., Cudeck R. (1992). Alternative ways of assessing model fit. Sociol Methods Res.

[65] Nudelman G., Otto K. (2021). Personal belief in a just world and conscientiousness: a meta-analysis, facet-level examination, and mediation model. Br J Psychol.

[66] Cipriani E., Frumento S., Gemignani A., Menicucci D. (2024). Personality traits and climate change denial, concern, and proactivity: a systematic review and meta-analysis. J Environ Psychol.

[67] Gregorich S.E. (2006). Do self-report instruments allow meaningful comparisons across diverse population groups? Testing measurement invariance using the confirmatory factor analysis framework. Med Care.

[68] (2024). https://www.destatis.de/DE/Themen/Gesellschaft-Umwelt/Bildung-Forschung-Kultur/Bildungsstand/Tabellen/bildungsabschluss.html;2020.

[69] (2023). https://www-genesis.destatis.de/genesis/online?operation=abruftabelleBearbeiten&levelindex=1&levelid=1713354892918&auswahloperation=abruftabelleAuspraegungAuswaehlen&auswahlverzeichnis=ordnungsstruktur&auswahlziel=werteabruf&code=12411-0003&auswahltext=&werteabruf=starten#abreadcrumb.

[70] Beiser-McGrath L.F., Bernauer T. (2021). Current surveys may underestimate climate change skepticism evidence from list experiments in Germany and the USA. Plos one.

[71] Organization World Health (May 2024). Strengthening mental health and psychosocial support before, during and after armed conflicts, natural and human-caused disasters and health and other emergencies. Seventy-seventh world health assembly. Agenda Item.

