# Corrigendum to “Development and validation of a climate change version of the man-made disaster-related distress scale (CC-MMDS)” [J Climate Change Health 20 (2024) 100356]

**DOI:** 10.1016/j.joclim.2025.100455

**Published:** 2025-05-09

**Authors:** Jil Beckord, Julia Barbara Krakowczyk, Nadja Gebhardt, Leonie Sophie Geiser, Katharina Kamler, Christoph Nikendei, Eva-Maria Skoda, Martin Teufel, Alexander Bäuerle

**Affiliations:** a Clinic for Psychosomatic Medicine and Psychotherapy, University of Duisburg-Essen, LVR-University Hospital Essen, Essen, Germany; b Center for Translational Neuro- and Behavioral Sciences (C-TNBS), University of Duisburg-Essen, Essen, Germany; c Clinic for General Internal Medicine and Psychosomatic, University Hospital Heidelberg, Heidelberg, Germany

The authors regret that the response scale of the CC-MMDS was accidentally mislabelled as “strongly disagree” and “strongly agree”, when instead it should have been labelled as “does not apply at all” and “applies fully”.

The mishap applies to the description of the scale in the Methods section 2.1 and to the German description in the supplement mmc1 in Appendix C (here, instead of “Ich stimme überhaupt nicht zu” and “Ich stimme voll und ganz zu”, it should be “trifft überhaupt nicht zu” and “trifft voll und ganz zu”).

The results and conclusions are not affected by this corrigendum.

The authors would like to apologise for any inconvenience caused.

