# Supplementary material for: Development and validation of a climate change version of the man-made disaster-related distress scale (CC-MMDS)
Source: J Clim Chang Health. 2024 Oct 18;20:100356. doi: 10.1016/j.joclim.2024.100356 (PMC12851373; doi:10.1016/j.joclim.2024.100356)
Supplement: Supplementary file 1 [file mmc1.docx]

**Appendix A**

**Table A.1 Original and translated items of the CC-MMDS**

In der letzten Woche… Over the past week…

| Item Nr. | Original item | Translated item |
| --- | --- | --- |
| 01 | Musste ich, auch ohne es zu beabsichtigen, an den Klimawandel denken. | I had to think about the climate change, even without intending to. |
| 02 | Habe ich mich im Zusammenhang mit dem Klimawandel ängstlich, besorgt oder nervös gefühlt. | I have felt anxious, worried, or nervous about the climate change. |
| 03 | Habe ich mich abgelenkt, um nicht an den Klimawandel zu denken. | I have distracted myself to avoid thinking about the climate change. |
| 04 | Fiel es mir schwer mich zu konzentrieren, wenn ich an den Klimawandel dachte. | I found it hard to concentrate when I thought about the climate change. |
| 05 | Fühlte ich mich bei dem Gedanken an den Klimawandel bedrückt. | I felt depressed at the thought of climate change. |
| 06 | Fühlte ich mich hilflos, wenn ich an den Klimawandel dachte. | I felt helpless when I thought about the climate change. |
| 07 | Fühlte ich mich schuldig, wenn ich an den Klimawandel dachte. | I felt guilty when I thought about the climate change. |
| 08 | Empfand ich Ärger oder Wut, wenn ich an den Klimawandel dachte. | I felt anger or rage when I thought about the climate change. |
| ~~09~~ | ~~Wünschte ich, der Klimawandel würde nicht existieren.~~ | ~~I wished the climate change did not exist.~~ |
| 10 | Hat das Ausmaß des Klimawandels mein Weltbild erschüttert. | The extent of the climate change has shaken my worldview. |
| 11 | Ließ mich der Klimawandel an der Menschheit zweifeln. | The climate change made me doubt mankind. |
| 12 | Ließ mich der Klimawandel an einer gerechten Welt zweifeln. | The climate change made me doubt a just word. |
| 13 | Zweifelte ich an dem politischen Umgang mit dem Klimawandel. | I had my doubts about the political approach to climate change. |
| 14 | Ließ mich der Klimawandel an gesellschaftlichen Normen und Werten zweifeln. | The climate change made me doubt social norms and values. |
| 15 | Hatte ich Angst vor zukünftigen negativen Folgen, welche durch den Klimawandel ausgelöst werden können. | I was afraid of future negative consequences, which could be triggered by the climate change. |
| 16 | Verspürte ich aufgrund des Klimawandels mehr Unsicherheit als sonst. | I felt more uncertainty than usual due to the climate change. |
| 17 | Fiel es mir, aufgrund des Klimawandels, zunehmend schwerer, positiv in die Zukunft zu blicken. | The climate change has made it increasingly difficult for me to look positively into the future. |

**Appendix B**

**Table B.1 Results of Item Analysis**

| Item | Discrimination | Difficulty | Mean | SD | α if deleted |
| --- | --- | --- | --- | --- | --- |
| CC-MMDS_01 | 0.62 | 0.74 | 5.17 | 1.88 | 0.94 |
| CC-MMDS_02 | 0.82 | 0.6 | 4.19 | 1.92 | 0.94 |
| CC-MMDS_03 | 0.61 | 0.39 | 2.7 | 1.92 | 0.94 |
| CC-MMDS_04 | 0.61 | 0.34 | 2.4 | 1.71 | 0.94 |
| CC-MMDS_05 | 0.82 | 0.64 | 4.45 | 1.98 | 0.94 |
| CC-MMDS_06 | 0.76 | 0.62 | 4.36 | 1.88 | 0.94 |
| CC-MMDS_07 | 0.52 | 0.49 | 3.41 | 1.75 | 0.94 |
| CC-MMDS_08 | 0.71 | 0.63 | 4.43 | 2.01 | 0.94 |
| CC-MMDS_09 | 0.46 | 0.8 | 5.57 | 1.9 | 0.94 |
| CC-MMDS_10 | 0.63 | 0.51 | 3.54 | 1.89 | 0.94 |
| CC-MMDS_11 | 0.7 | 0.69 | 4.83 | 1.86 | 0.94 |
| CC-MMDS_12 | 0.7 | 0.7 | 4.88 | 1.94 | 0.94 |
| CC-MMDS_13 | 0.6 | 0.83 | 5.8 | 1.43 | 0.94 |
| CC-MMDS_14 | 0.63 | 0.71 | 4.97 | 1.76 | 0.94 |
| CC-MMDS_15 | 0.8 | 0.73 | 5.14 | 1.84 | 0.94 |
| CC-MMDS_16 | 0.76 | 0.56 | 3.92 | 2.02 | 0.94 |
| CC-MMDS_17 | 0.77 | 0.61 | 4.26 | 1.99 | 0.94 |

*Mean inter-item-correlation = 0.487 · Cronbach's α = 0.942*


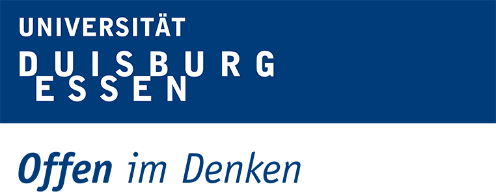

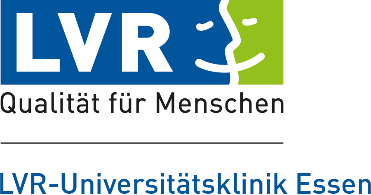


**Appendix C**

**Table C.1 Climate Change – Man-Made Disaster-Related Distress Scale (CC-MMDS)**

*1 = Ich stimme überhaupt nicht zu*

*7 = Ich stimme voll und ganz zu*

In der letzten Woche…

| 1 | Musste ich, auch ohne es zu beabsichtigen, an den Klimawandel denken. |  |  |  |  |  |  |  |
| --- | --- | --- | --- | --- | --- | --- | --- | --- |
| 2 | Habe ich mich im Zusammenhang mit dem Klimawandel ängstlich, besorgt oder nervös gefühlt. |  |  |  |  |  |  |  |
| 3 | Habe ich mich abgelenkt, um nicht an den Klimawandel zu denken. |  |  |  |  |  |  |  |
| 4 | Fiel es mir schwer mich zu konzentrieren, wenn ich an den Klimawandel dachte. |  |  |  |  |  |  |  |
| 5 | Fühlte ich mich bei dem Gedanken an den Klimawandel bedrückt. |  |  |  |  |  |  |  |
| 6 | Fühlte ich mich hilflos, wenn ich an den Klimawandel dachte. |  |  |  |  |  |  |  |
| 7 | Fühlte ich mich schuldig, wenn ich an den Klimawandel dachte. |  |  |  |  |  |  |  |
| 8 | Empfand ich Ärger oder Wut, wenn ich an den Klimawandel dachte. |  |  |  |  |  |  |  |
| 10 | Hat das Ausmaß des Klimawandels mein Weltbild erschüttert. |  |  |  |  |  |  |  |
| 11 | Ließ mich der Klimawandel an der Menschheit zweifeln. |  |  |  |  |  |  |  |
| 12 | Ließ mich der Klimawandel an einer gerechten Welt zweifeln. |  |  |  |  |  |  |  |
| 13 | Zweifelte ich an dem politischen Umgang mit dem Klimawandel. |  |  |  |  |  |  |  |
| 14 | Ließ mich der Klimawandel an gesellschaftlichen Normen und Werten zweifeln. |  |  |  |  |  |  |  |
| 15 | Hatte ich Angst vor zukünftigen negativen Folgen, welche durch den Klimawandel ausgelöst werden können. |  |  |  |  |  |  |  |
| 16 | Verspürte ich aufgrund des Klimawandels mehr Unsicherheit als sonst. |  |  |  |  |  |  |  |
| 17 | Fiel es mir, aufgrund des Klimawandels, zunehmend schwerer, positiv in die Zukunft zu blicken. |  |  |  |  |  |  |  |


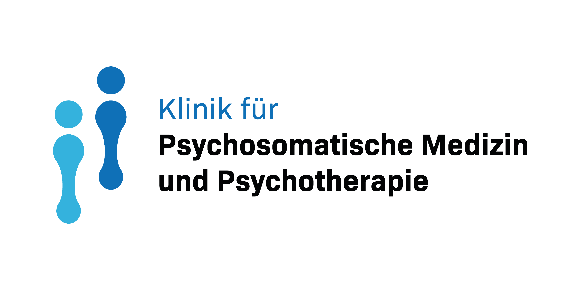


**Appendix D**

**Table D.1 Factor loadings of the CC-MMDS items.**

| Item Nr. | Items of the CC-MMDS |  | Factor loadings on Psychological distress | Factor loadings on Change of existing belief systems |
| --- | --- | --- | --- | --- |
| 01 | I had to think about the climate change, even without intending to. |  | 0.50 |  |
| 02 | I have felt anxious, worried, or nervous about the climate change. |  | 0.86 |  |
| 03 | I have distracted myself to avoid thinking about the climate change. |  | 0.80 |  |
| 04 | I found it hard to concentrate when I thought about the climate change. |  | 0.86 |  |
| 05 | I felt depressed at the thought of climate change. |  | 0.80 |  |
| 06 | I felt helpless when I thought about the climate change. |  | 0.68 |  |
| 07 | I felt guilty when I thought about the climate change. |  | 0.45 |  |
| 08 | I felt anger or rage when I thought about the climate change. |  | 0.36 | 0.43 |
| 10 | The extent of the climate change has shaken my worldview. |  | 0.41 |  |
| 11 | The climate change made me doubt mankind. |  |  | 0.93 |
| 12 | The climate change made me doubt a just word. |  |  | 0.91 |
| 13 | I had my doubts about the political approach to climate change. |  |  | 0.71 |
| 14 | The climate change made me doubt social norms and values. |  |  | 0.75 |
| 15 | I was afraid of future negative consequences, which could be triggered by the climate change. |  | 0.52 | 0.37 |
| 16 | I felt more uncertainty than usual due to the climate change. |  | 0.75 |  |
| 17 | The climate change has made it increasingly difficult for me to look positively into the future. |  | 0.62 |  |

*Cut = .3*
