# Supplementary material for: Development and validation of a climate change version of the man-made disaster-related distress scale (CC-MMDS)
Source: J Clim Chang Health. 2024 Oct 18;20:100356. doi: 10.1016/j.joclim.2024.100356 (PMC12851373; doi:10.1016/j.joclim.2024.100356)
Supplement: Supplementary file 2 [file mmc2.docx]

Submission Requirements The Journal of Climate Change and Health:

Note to authors: This form must be completed in full and uploaded with your submission: incomplete answers will result in delay in review of your submission and return to the author for correction

I have read the Guide for Authors and followed its recommendations

The cover letter is complete and includes

The type of article

Whether it is intended for a Special Issue

The word count for text (not including references, figures, tables or abstract)

The abstract is limited to 250 words

The abstract abides by the following standards (check one)

Research Articles and Reviews require a structured abstract with Introduction, Methods, Results, and Conclusions

Short Communications, Case Reports, and Perspectives require an unstructured abstract.

The article, according to the standards described in the Guide for Authors, follows the standards based on article type for word count, reference number and format including numbering of sections

In-text citations are bracketed and in Arabic numerals [ ]

In-text citations come before punctuation at the end of a sentence

Language/grammar has been checked by a primary English speaker if you are not a primary English-speaker

Language/grammar is in American English

A potential reviewer is listed with their email address

References are complete/accurate, in Vancouver format and in accordance with the

Journal of Climate Change and Health’s information to authors

All references in the reference list are cited in the text?

The conflict of interest form titled “The Declaration Tool” completely filled out?
